# Establishment of a murine epidermal cell line suitable for *in vitro *and *in vivo *skin modelling

**DOI:** 10.1186/1471-5945-11-9

**Published:** 2011-04-21

**Authors:** Carmen Segrelles, Almudena Holguín, Pilar Hernández, José M Ariza, Jesús M Paramio, Corina Lorz

**Affiliations:** 1 Molecular Oncology Unit, Epithelial Biomedicine Division, Basic Research Department, Centro de Investigaciones Energéticas, Medioambientales y Tecnológicas (CIEMAT), Avd. Complutense 22, Madrid 28040, Spain; 2 Regenerative Medicine Unit, Epithelial Biomedicine Division, Basic Research Department, Centro de Investigaciones Energéticas, Medioambientales y Tecnológicas (CIEMAT), Avd. Complutense 22, Madrid 28040, Spain

## Abstract

**Background:**

Skin diseases are a major health problem. Some of the most severe conditions involve genetic disorders, including cancer. Several of these human diseases have been modelled in genetically modified mice, thus becoming a highly valuable preclinical tool for the treatment of these pathologies. However, development of three-dimensional models of skin using keratinocytes from normal and/or genetically modified mice has been hindered by the difficulty to subculture murine epidermal keratinocytes.

**Methods:**

We have generated a murine epidermal cell line by serially passaging keratinocytes isolated from the back skin of adult mice. We have termed this cell line COCA. Cell culture is done in fully defined media and does not require feeder cells or any other coating methods.

**Results:**

COCA retained its capacity to differentiate and stratify in response to increased calcium concentration in the cell culture medium for more than 75 passages. These cells, including late passage, can form epidermis-like structures in three-dimensional *in vitro *models with a well-preserved pattern of proliferation and differentiation. Furthermore, these cells form epidermis in grafting assays *in vivo*, and do not develop tumorigenic ability.

**Conclusions:**

We propose that COCA constitutes a good experimental system for *in vitro *and *in vivo *skin modelling. Also, cell lines from genetically modified mice of interest in skin biology could be established using the method we have developed. COCA keratinocytes would be a suitable control, within a similar background, when studying the biological implications of these alterations.

## Background

Skin diseases affect patients of all ages worldwide. By some estimates, they affect 50 percent or more of the population at any one time and comprise over 2,000 medical conditions which can range from only mild skin problems to pathologies that are serious or even fatal. The aetiology of skin diseases is varied and some of the most severe are autoimmune and genetic disorders, including cancer.

Skin tissue engineering techniques emerged in the 1980s to address the need for extensive full-thickness burns coverage in the absence of sufficient autologous skin for grafting [1-3]. In the clinical context they are also useful in treating non-healing ulcers [4,5]. In addition to its clinical applications, three-dimensional models (3D) of engineered skin are being broadly used in skin biology research, such as reducing animal experimentation (i.e. animal testing for human skin products), investigation of cell interactions and skin barrier penetration, and the development of models of human skin diseases, such as psoriasis [6-8] and the genetic disorder epidermolysis bullosa [9,10].

Over the last years, genetically modified (transgenic and/or null) mouse models have been developed that recapitulate to a variable extent human diseases, including skin disorders. There is a lot to learn from engineered epidermis using keratinocytes from these animal models. However, despite the early success in culturing human keratinocytes [11], primary murine keratinocytes have shown to be fairly reluctant to subculture and expansion *in vitro*, thus precluding the establishment of reproducible 3D models to be used in skin biology research. Initial attempts to culture murine keratinocytes long-term required the use of matrix coated dishes and/or complex fibroblast-conditioned media with fetal bovine serum for cell seeding and growth [12-14]. These cells retained their ability to differentiate, when the concentration of calcium in the culture media was increased, only until passage 10 [12] to 15 [14]. Caldelari *et al*. [15] and Reichelt & Haase [16] achieved serial subculture of murine epidermal keratinocytes for more than 50 and 250 passages, respectively. While Reichelt & Haase method still required the use of feeder cells in the initial phases of growth, collagen-coated dishes and serum-conditioned culture media, Caldelari *et al*. [15] subcultured cells in uncoated culture dishes and fully defined media. Also, they showed that their cell line retained the capacity to respond to the elevation of calcium concentration in the culture media with the establishment of intercellular adhesion complex and the expression of terminal differentiation markers.

There has been great advance in the development of fully defined media to culture keratinocytes and to establish 3D epidermal models; however, theses advances are being focused and tested on primary human epidermal keratinocytes. In fact, when compared to the field of human tissue-engineered skin (for a review [17]), current experience on 3D models using cultured murine keratinocytes is scarce, consisting of organotypic cultures that use devitalised dermis and complex non-defined culture media [13,18].

We have established a murine keratinocyte cell line that grows on conventional cell culture plastic dishes and with defined culture media. These cells retain its potential to differentiate for more than 75 passages when the calcium concentration is increased, and form an epidermis-like structure using the abovementioned 3D protocols. This murine cell line, termed COCA, is non-tumorigenic and can also form an epidermis *in vivo *when grafted onto immunodeficient mice. We propose that our cell culture technique is suitable for the establishment of other murine cell lines bearing different genetic manipulations and that these cell lines can be used to establish reproducible 3D epidermal models appropriate for skin biology research.

## Methods

### Cell isolation and culture

Keratinocytes were isolated from the back skin of 2-months old C57BL/DBA mice using a protocol suitable for adult skin [19]. They were maintained in culture in a low calcium (0.07 mM) progenitor cell targeted fully defined media, CnT-07 (CELLnTEC, Bern, Switzerland), in an incubator at 37°C and 5% CO_2_. At confluency, cells were subcultured by trypsinisation and plated at 4.5-5.5 × 10^3 ^cells per cm^2 ^in seeding media (EMEM, 4% FBS pre-treated with Chelex 100 resin, 0.2 mM CaCl_2_) for 8-14 hours to allow cell adhesion. Then, culture plates were washed twice with PBS to eliminate calcium, and switched to CnT-07 to promote cell growth and prevent differentiation. FBS-seeding media is not further needed once the cell line has been established (>20 passages). At this point, 8-14 hours incubation in CnT-07 medium containing 0.2 mM CaCl_2 _is enough to ensure good cell attachment after trypsinisation.

Conventional *in vitro *keratinocyte differentiation was initiated when cells were confluent by changing the low calcium (0.07 mM) media to a high calcium (1.2 mM) differentiation media. This elevation in the concentration of calcium induces the cells to differentiate [20-22]. Bromodeoxyuridine 10 μM (BrdU) (Sigma Aldrich, St. Louis, MO) was added to the culture medium 1 hour before sample fixation.

Three-dimensional (3D) *in vitro *epidermal cell cultures were established by culturing COCA in CELLnTEC fully defined cell culture media following the manufacturer's recommendations, in the absence of fibroblasts or other supporting cells. Briefly, 200,000 cells were seeded into Millipore PCF 0.4 μm inserts placed in a Petri dish. Cells were grown for four days in CnT-07, and switched to CnT-02-3D when they reached confluency. Culture media in and outside the inserts was left for an overnight; then, culture media inside the inserts was aspirated to start the stratification process. This system creates an air/liquid interface that allows the formation of a multilayered structure with a population of proliferative cells in the basal layer and a differentiated progeny. CnT-02-3D media was changed every 2-3 days. Development of 3D epidermal cell cultures was followed for 3 weeks.

### Mice

All the animal work was approved by the Animal Ethical Committee (CEEA) and conducted in compliance with Centro de Investigaciones Energéticas, Medioambietales y Tecnológicas (CIEMAT) guidelines. NMRI nude (nu/nu) immunodeficient 5-week old female mice were obtained from Janvier (Saint-Berthevin, France) and housed in our animal facilities for 1-2 weeks prior to any experimental procedures. Mice were injected intraperitoneally with 100 μg of BrdU (dissolved in 0.09% NaCl) per g of body weight 1 hour before they were sacrificed to analyse proliferation (BrdU incorporation).

### Tumorigenicity assay

To assay tumour-formation ability, 1 × 10^6 ^COCA cells in late passages (>70) were resuspended in 100 μl saline buffer and injected subcutaneously on the flanks of NMRI nude mice. Both flanks were used, in one COCA were injected alone, in the other they were combined with 0.5 × 10^6 ^dermal fibroblasts. The experiment was repeated three times using 5 mice (x2 flanks) per experiment. Tumour growth was not evident, and animals were sacrificed and studied at 4-6 months to search for any indication of tumour.

### Cell grafting assay

To assay the ability of COCA cells to engraft we followed a protocol adapted from Strachan and Ghadially [23]. Briefly, mice were anesthetised and an excision approximately 5 mm in length was performed in the dorsal skin just between the shoulder blades. Silicone chambers with a 7 mm internal diameter (Renner, Dannstadt, Germany) were implanted and sutured in place when required. COCA epidermal keratinocytes (1.5 × 10^6^) in passage 77 were mixed with dermal fibroblasts (0.5 × 10^6^), resuspended in a final volume of 100 μl PBS and injected into the silicon chambers. The cells were allowed 20 min to settle and adhere before waking up the mice. Epidermis formed by grafted COCA + fibroblasts was harvested 4 weeks after chamber implantation. Mice were injected i.p. with 100 μg/g of body weight BrdU 1 h before they were sacrificed to analyse proliferation.

### Histological procedures

For histological analysis, cells and tissue samples were fixed in 4% paraformaldehyde and embedded in paraffin prior to sectioning. Sections 3-4 μm thick were stained and processed as described [24]. For BrdU detection, prior to blocking with serum, samples were incubated for 30 min in 1 N HCl (cells) or 1 h in 2 N HCl (tissue). Primary antibodies used were rabbit polyclonal anti-K5, anti-K6, Loricrin, Filaggrin (Covance, Emeryville, CA), and anti-BrdU (AbCam, Cambridge, UK); and mouse monoclonal anti-K10 (for cells clone RKSE60, St. Cruz Biotechnology, Santa Cruz, CA; for tissue sections DE-K10, DakoCytomation, Denmark A/S) and anti-PCNA (clone PC10 Thermo Scientific, Fremount, CA). FITC or TexasRed conjugated secondary antibodies were purchased from Jackson ImmunoResearch (West Grove, PA). Control slides were obtained by replacing primary antibodies with PBS or preimmune sera.

## Results

### Establishment of a long-term culture of murine epidermal keratinocytes

Keratinocytes were isolated from the back skin of 2-months old C57BL/DBA mice and maintained in culture in low calcium (0.07 mM) fully defined media (CnT-07). Cells were repeatedly passaged to establish a cell line. We termed this murine epidermal keratinocyte derived cell line COCA, and we have expanded it in culture for more than 80 passages. COCA cell line has been deposited (October 2010) under the reference 10112001 with ECACC (European Collection of Cell Cultures, http://www.hpacultures.org.uk/products/celllines/generalcell/detail.jsp?refId=10112001&collection=ecacc_gc).

Cytogenetic studies using the spectral karyotype (SKY) (Table 1 and Additional file 1) revealed that a translocation between chromosomes 2 and 4 occurred early (already present in passage 8 cells) and is maintained. Murine epidermal keratinocytes in culture have a tendency to became tetraploid [14,18]; however, COCA showed to be genetically more stable than previously published cell lines [18] and was still diploid at passage 8 (Table 1).

**Table 1:** Cytogenetic study (SKY) of COCA epidermal keratinocytes at different passages.

Passage n°	Metaphases analysed	Chromosome n°		Chromosome rearrangements	Sex
				
		Range	Modal number		
8	10	38-43	40	der(4)T(2;4)	XX
52	15	64-77	71	der(4)T(2;4)	XXXX
77	15	67-79	75	der(4)T(2;4) + 2 centromeric fagments	XXX

T: translocation.

### Formation of conventional and 3D epidermal differentiation *in vitro *models

The epidermis is a multilayered epithelium where the process of terminal differentiation takes place as the committed cells in the basal proliferative compartment arrest proliferation and move upwards towards the epidermal surface. This process is accompanied by changes in the expression of keratins and other proteins. Keratin 5 (K5) is expressed in the basal layer, where proliferation occurs, and marks undifferentiated cells. Once the cells start the differentiation program they cease the expression of K5 and start the synthesis of the keratin K10, which is considered as an early marker of differentiation *in vivo *and *in vitro*. Terminal differentiation is indicated by the expression of loricrin and filaggrin. The keratin K6 is induced in the interfollicular epidermis as consequence of inflammatory and/or hyperproliferative stimuli, and in many circumstances this is in parallel with reduced K10 expression and expansion of K5 to suprabasal layers.

**Figure 1:**
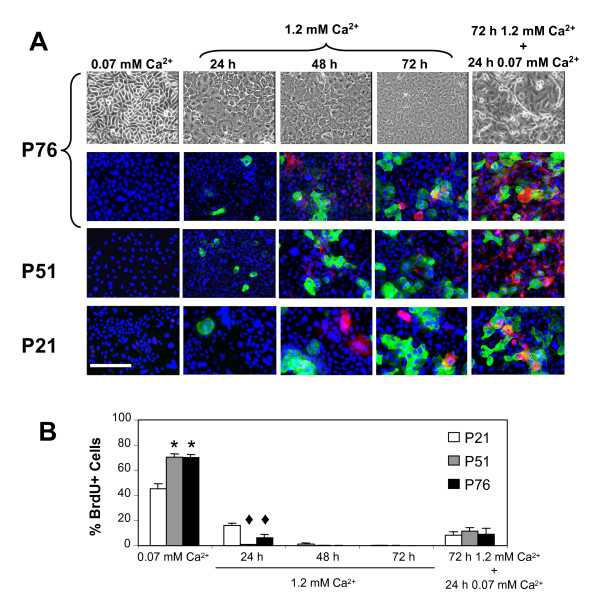
**Differentiation of COCA murine epidermal keratinocytes**. A) Phase contrast micrographs (upper panel) showing COCA murine keratinocytes from passage 76 (P, denotes passage) growing in low (0.07 mM) or high (1.2 mM) calcium medium at the indicated times, and re-stimulated to grow with low calcium medium for an additional 24 h. Expression of differentiation markers was studied by immunofluorescence. In blue, nuclei stained with DAPI; in green, the early differentiation marker K10; in red, the terminal differentiation marker loricrin. Scale bar: 200 μm. All micrographs were taken at the same scale. B) Percentage of BrdU incorporation in keratinocytes growing under the indicated conditions. At least 1,000 cells from 5-10 different fields were scored in each time point (mean ± standard error). Student's t-test; * p < 0.05 vs. P21 in 0.07 mM Ca^2+^; ♦ p < 0.05 vs. P21 in 24 h 1.2 mM Ca^2+^.

Keratinocytes can be induced to differentiate *in vitro *by increasing the concentration of calcium in the culture medium [20-22]. To evaluate the potential of COCA to differentiate we replaced the low calcium medium (0.07 mM) for a differentiation medium containing a high concentration of calcium (1.2 mM). As early as 24 hours in 1.2 mM calcium differentiation medium, keratinocytes start to flatten (Figure 1A, upper row panels) and to establish cell-cell contacts. Upon calcium elevation, COCA mouse keratinocytes in all passages studied (passage 21, 51 and 76), displayed an arrest in proliferation (Figure 1B) and a similar pattern of differentiation marker expression: K10 expressing cells are detected at 24-48 hours and loricrin positive cells at 48-72 hours in differentiation media (Figure 1A, immunofluorescence panels). Cells from passage 51 and 76 growing in low calcium medium displayed an increased proliferation rate as compared to that of passage 21, this could be due to further adaptation of the cells occurring between passage 21 and 51. Still, cells at all passages tested readily responded with an arrest in proliferation when switched for 24 hours to a high calcium media. Most importantly, after 72 hours in differentiation medium, cells were re-stimulated for 24 hours in the presence of low calcium growth medium and, independently of the passages studied, expression of terminal differentiation markers was achieved and BrdU incorporation was minimal, demonstrating that differentiation is associated to permanent cell cycle withdrawal (Figure 1A, right column panels, and Figure 1B).

**Figure 2:**
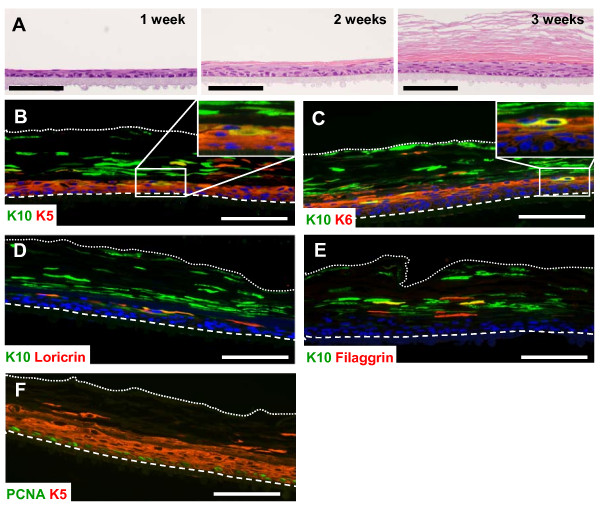
**Three-dimensional *in vitro *epidermal cell cultures using COCA keratinocytes**. A) H&E staining showing the formation of a 3D *in vitro *epidermal cell culture using passage 76 COCA keratinocytes. The micrographs show the progression of the culture for 1, 2 and 3 weeks after inducing the cells to differentiate by creating an air/liquid interface. B-F) Immunofluorescence staining of 3 week 3D cultures. B) K5 expression is expanded to suprabasal layers reflecting the hyperproliferative nature of the model (for details see inset). C) K6 is expressed mostly in suprabasal layers (for details see inset). B-E) K10 is expressed in suprabasal layers and some K10 positivity can be detected in the corneous stratum. Loricrin (D) and filaggrin (E) are also expressed in suprabasal layers. PCNA staining (F) showed that proliferation is mostly restricted to the basal layer cells. Dashed lines mark the contact of the basal layer of the 3D culture with the PCF membrane. Dotted lines mark the end of the corneous stratum. Scale bars: 100 μm.

Although useful, the above described *in vitro *differentiation model has important limitations; in this sense, 3D models resemble the *in vivo *situation much more closely. In these systems, the keratinocytes are organised in a multiple layered structure, with a proliferating basal layer that stratifies as it matures and differentiates. We have followed a 3D epidermal *in vitro *model that avoids the use of supporting cells, collagen matrix and non-defined cultured media. Cell cultures of COCA in passage 76 were induced to differentiate by creating an air/liquid interface. After three weeks a full epidermis-like structure was formed (Figure 2A). K5 was expressed in basal and suprabasal layer cells while K6 and K10 were only expressed by suprabasal layers (Figure 2B-E). Suprabasal layers express the terminal differentiation markers loricrin and filaggrin (Figure 2D&2E). Proliferating cell nuclear antigen (PCNA) staining shows that the layer of proliferating cells that maintain the architecture of the culture locates at the base (Figure 2F). Limitations to this 3D model affect K10 expression that is patched rather that continuous, as well as that of the terminal differentiation markers loricrin and filaggrin. Also, the normal sequence of expression of these markers is not fully preserved.

### COCA keratinocytes are non-tumorigenic and regenerate epidermis in *in vivo *assays

To explore whether prolonged culture conditions might have conferred tumorigenic potential to COCA cells we performed *in vivo *tumorigenicity assays using immunodeficient mice [25-27]. In this assays, keratinocytes were injected in the flanks of nu/nu mice alone or mixed with dermal fibroblasts to help sustain keratinocyte growth. After 4-6 months there was no external sign of tumour growth and animals were sacrificed and studied to search for any evidence of tumour formation. Experiments using genetically modified murine keratinocytes were done in parallel and yielded 100% tumour growth by 2 months, both in the presence/absence of dermal fibroblasts (data not shown).

**Figure 3:**
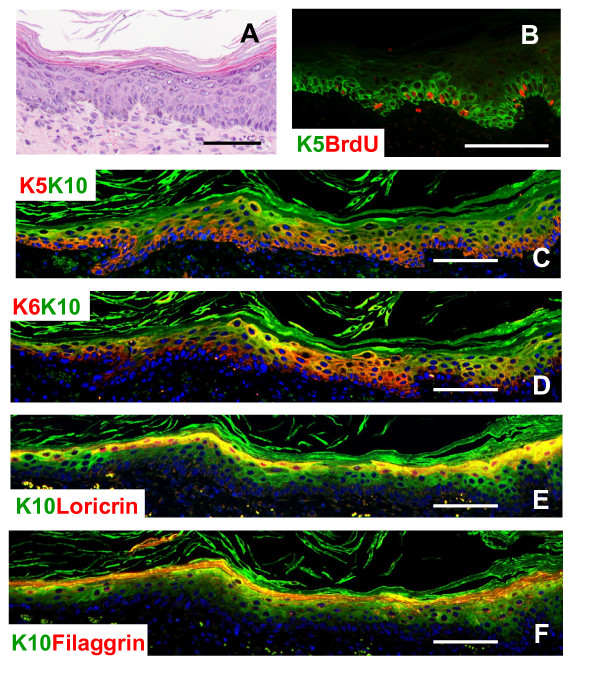
**COCA keratinocytes regenerate an epidermis-like structure *in vivo *upon grafting**. COCA keratinocytes in passage 77 were grafted into silicon chambers placed in the back skin of immunodeficient mice. H&E (A) and immunofluorescence (B-F) staining of the epidermis formed 4 weeks after grafting. B&C) K5 was expressed mainly by basal keratinocytes but displayed some expansion to suprabasal layers; however, BrdU-K5 positive cells were located mainly in the basal layer (B). D) K6 was present in basal and suprabasal layers and overlapped partially with the expression of the early differentiation marker K10. C-F) K10 was not expressed in the basal layer. Loricrin (E) and filaggrin (F) were expressed in suprabasal layers. Scale bars: 100 μm.

To rule out the possibility that subcutaneous injection is an adverse environment for non-genetically modified keratinocytes to thrive we used a different approach, silicone chambers implanted onto the back of immunodeficient mice which might provide a more physiological environment for the keratinocytes to grow. In these grafting assays, keratinocytes are seeded on the subcutaneous fascia of immunodeficient mice with dermal fibroblasts. After 1 month of grafting, COCA keratinocytes generated a fully differentiated epidermis-like structure (Figure 3A). This epidermis displays some hyperplasia due to increased proliferation as demonstrated by the expansion of K5 to suprabasal layers, K6 expression and high rate of BrdU incorporation (Figure 3B, C &3D). However, expression of early and late differentiation markers is maintained (K10 in Figure 3C-F, loricrin in 3E & filaggrin in 3F). No signs of dysplasia or malignant transformation were evident. When the experiments were done grafting genetically modified keratinocytes, papillomas with evident signs of malignant transformation developed (data not shown).

## Discussion

Three-dimensional models represent the most accurate method of modelling skin complex biological processes *in vitro*. Also, genetically modified mouse models have a lot to offer in the study of skin disease research. However, the difficulties to establish long-term cultures of murine epidermal keratinocytes have hindered the development of murine 3D cultures.

We have established a non-transformed murine keratinocyte cell line that grows on conventional cell culture plastic dishes without the need of feeder cells or coating matrix. Also, cell growth is done in easy-to-use commercially available defined cell culture media. We have termed this murine keratinocyte cell line COCA and we show its capacity to grow in 3D *in vitro *models and to reconstitute epidermis in cell grafting assays *in vivo*.

In agreement with previous studies [14,18], COCA keratinocytes show a tendency to tetraploidy when subcultured. SKY analyses revealed that while at passage 8 cells were still diploid, at passage 77 they were almost tetraploid. However, COCA cells seem more stable than previously reported cells that were nearly tetraploid after 8 to 10 passages [18]. This could be partly due to improved cell culture conditions or to the fact that COCA cells were established from adult mice keratinocytes instead from newborn pups. SKY analyses also revealed a clonal translocation between chromosomes 2 and 4 that occurred early in the culture.

All the same, these alterations in chromosome number are not linked to defects in differentiation. When induced to differentiate by raising the calcium concentration in the culture media, COCA cells expressed the early differentiation-specific keratin K10 and the terminal differentiation marker loricrin, with timing and pattern conserved in early throughout late passages. In the skin, keratinocyte differentiation is accompanied by a permanent arrest in proliferation. Similarly, COCA cells arrest their proliferation when induced to differentiate by calcium and they hardly re-enter in cell cycle when stimulated again with low calcium growth medium [28]. This is indicative of a postmitotic terminally differentiated cell, characteristic of differentiated keratinocytes *in vivo*.

Next, we showed that COCA are also able to establish 3D models where keratinocytes are induced to differentiate by creating an air/liquid interface. Even late passage COCA keratinocytes (>75) are able to form an organised epidermis-like structure where proliferating cells are situated in the basal layer and K10, loricrin and filaggrin expressing differentiated cells locate at suprabasal layers. There is an expansion in the expression of the basal keratinocyte K5 to suprabasal layers, and expression of K6, this could be indicative of increased proliferation. Also, some alterations are present in the normal sequence of differentiation marker expression. Of note, these alterations affecting the expression of differentiation markers K10, loricrin and filaggrin, were not observed in *in vivo *experiments, pointing out to a possible experimental limitation of the *in vitro *model. However, work done in our laboratory with keratinocytes from murine models bearing different genetic alterations show that these 3D *in vitro *models are useful to study some of the characteristics and behaviour of these cells (not shown).

*In vivo *assays using immunodeficient mice showed that COCA cells are non-tumorigenic. This was also supported by cell grafting assays where late passage COCA keratinocytes were capable of growing a well-preserved epidermal-like structure with no evident signs of tumoral alterations.

## Conclusions

We have generated an epidermal murine keratinocyte cell line, termed COCA. We show that COCA keratinocytes can establish reproducible 3D models applicable to the study of skin biology. The method we have followed to obtain and culture this cell line can be used to successfully develop keratinocyte cell lines from mice with genetic modifications of interest in the field of skin disease. Also, COCA could be a good control when studying the biological implications of these alterations.

## Competing interests

The authors declare that they have no competing interests.

## Authors' contributions

CS designed and performed the experiments. AH helped with grafting assays. PH performed the histology work. JMA did some of the immunostaining. JMP is Head of the Molecular Oncology Unit and advised on experiment design and manuscript writing. CL designed and performed the experiments, and wrote the manuscript. All authors have read and approved the final manuscript.

## Pre-publication history

The pre-publication history for this paper can be accessed here:

http://www.biomedcentral.com/1471-5945/11/9/prepub

## Supplementary Material

Additional file 1 **SKY figures of representative metaphases from COCA samples at the indicated passage numbers (P)**. Cells display a stable translocation between chromosomes 2 and 4 (T(2;4)), and are nearly tetraploid at P77. Click here for file
