# Supplementary figures and images for: Establishment of a murine epidermal cell line suitable for in vitro and in vivo skin modelling
Source: BMC Dermatol. 2011 Apr 21;11:9. doi: 10.1186/1471-5945-11-9 (PMC3113952; doi:10.1186/1471-5945-11-9)

## Slide 1
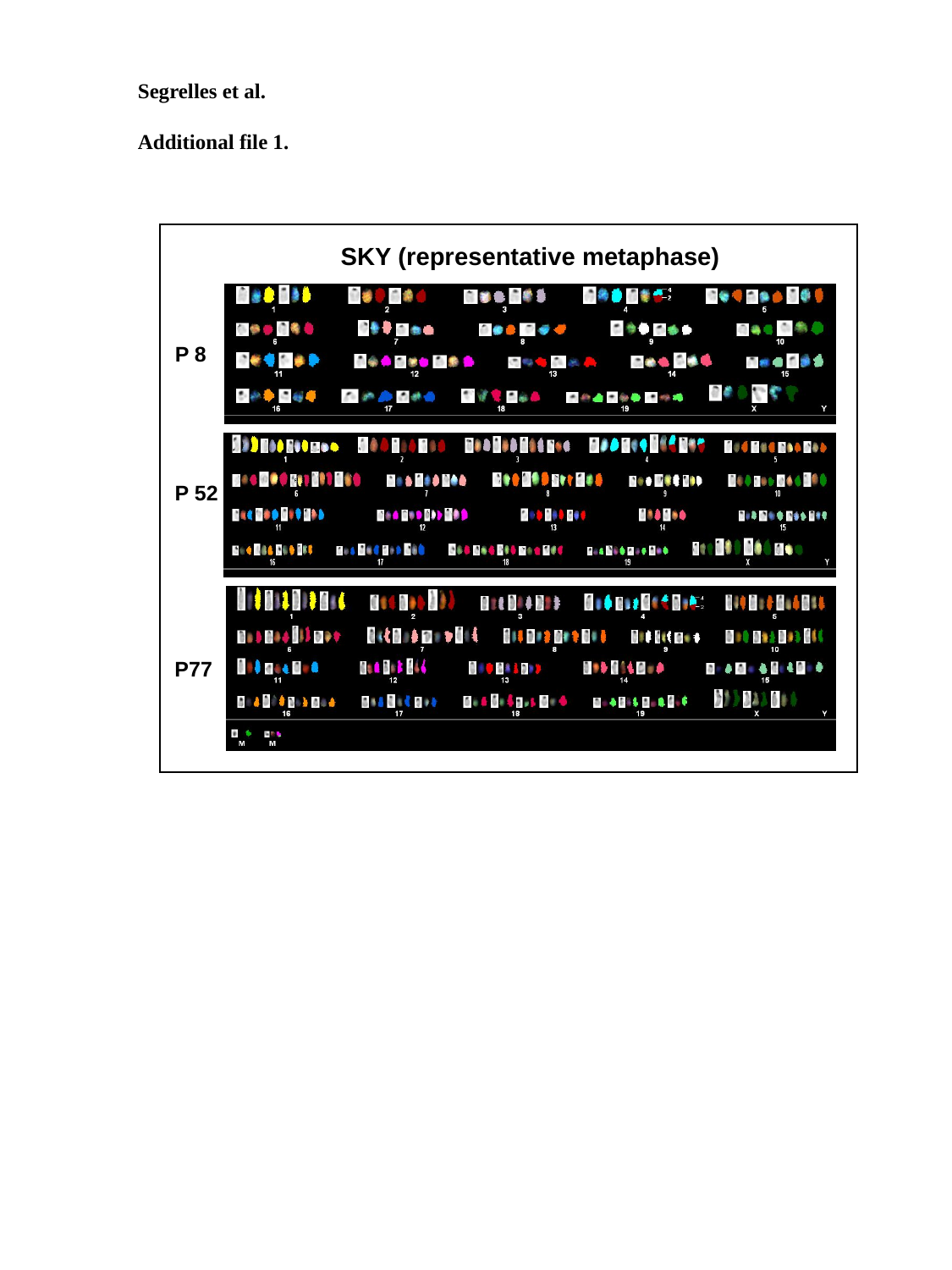

Segrelles et al.
Additional file 1.
SKY (representative metaphase)
P 8
P 52
P77

Supplement: Additional file 1 — SKY figures of representative metaphases from COCA samples at the indicated passage numbers (P). Cells display a stable translocation between chromosomes 2 and 4 (T(2;4)), and are nearly tetraploid at P77. [file 1471-5945-11-9-S1.PPT]
